# ParallelEvolCCM: Quantifying Coevolutionary Patterns Among Genomic Features

**DOI:** 10.1093/gbe/evaf092

**Published:** 2025-05-17

**Authors:** Robert G Beiko, Chaoyue Liu, João Vitor Cavalcante, Ryan C Fink

**Affiliations:** Faculty of Computer Science and Institute for Comparative Genomics, Dalhousie University, 6050 University Avenue, Halifax, Nova Scotia B3H 4R2, Canada; Faculty of Computer Science and Institute for Comparative Genomics, Dalhousie University, 6050 University Avenue, Halifax, Nova Scotia B3H 4R2, Canada; Department of Mathematics and Statistics, Dalhousie University, Halifax, Nova Scotia B3H 4R2, Canada; Bioinformatics Multidisciplinary Environment, Federal University of Rio Grande do Norte, Natal, Rio Grande do Norte 59076-550, Brazil; Faculty of Computer Science and Institute for Comparative Genomics, Dalhousie University, 6050 University Avenue, Halifax, Nova Scotia B3H 4R2, Canada

**Keywords:** phylogenetic proles, coevolution, phylogenetics

## Abstract

Concerted gains and losses of genomic features such as genes and mobile genetic elements can provide key clues into related functional roles and shared evolutionary trajectories. By capturing phylogenetic signals, a coevolutionary model can outperform comparative methods based on shared presence and absence of features. We previously developed the Community Coevolution Model, which represents the gain/loss probability of each feature as a combination of its own intrinsic rate, combined with the joint probabilities of gain and loss with all other features. Originally implemented as an R library, we have now developed an R wrapper that adds parallelization and several options to pre-filter the features to increase the efficiency of comparisons. Here we describe the functionality of ParallelEvolCCM and apply it to a dataset of 1000 genomes of the genus *Bifidobacterium*. ParallelEvolCCM is released under the MIT license and available at https://github.com/beiko-lab/arete/blob/master/bin/ParallelEvolCCM.R.

**Significance**

Patchy phylogenetic distributions of genes, mobile genetic elements, and other genomic features can constitute evidence for lateral gene transfer. Comparing the presence/absence patterns of multiple features can reveal important associations among them, but phylogenetic relationships must be taken into consideration in order to avoid spurious correlations. Our new ParallelEvolCCM software embeds these comparisons in a coevolutionary framework, offers a range of options to optimize the speed and comparisons, and offers helper scripts to visualize relationships among features.

## Introduction

The distribution of genomic features such as mutations, genes, and mobile genetic elements can be represented using presence/absence representations often referred to as phylogenetic profiles (Gaasterland and Ragan 1998; Pellegrini et al. 1999). Correlations and anticorrelations between these profiles can reveal a great deal about the capabilities, evolutionary history, and ecological roles of features and the implicated genomes. Although the term “phylogenetic” is present in their name, these profiles do not account for the evolutionary relationships among their genomes. In many applications, this can lead to misleading patterns of correlation, especially in datasets with highly uneven sampling, such as those involving clinically important pathogens. Indeed, the unevenness of sampling across the bacterial tree is acute in references such as the database constructed from over 661,000 reference bacterial genomes, where the 20 most-abundant species (all with high representation of human pathogenic isolates) comprise over 90% of all sequenced genomes (Blackwell et al. 2021). In these cases and in presence of high rates of lateral gene transfer among microorganisms, consideration of phylogenetic relationships can identify shared presence/absence patterns that are unexpected when compared to the reference tree of genomes. Several recent methods have aimed to correct for phylogenetic correlation in different ways including an approach based on Pagel’s coevolutionary model (Liu et al. 2018), PhyloCorrelate (Tremblay et al. 2021), and a method that uses the inverse Potts model to minimize the impact of spurious transitive correlations among features (Fukunaga and Iwasaki 2022).

Liu et al. (2023) introduced EvolCCM, an algorithm and R library that uses a coevolutionary model to identify correlation patterns among features by modeling each feature’s rate of change in terms of its own intrinsic rate, combined with interactions with other features in the set. We demonstrated the accuracy of EvolCCM on simulated profiles with different degrees of association and recovered key functional associations including shared GO terms and functional complexes of proteins. Although EvolCCM showed high levels of sensitivity, the inference of associations for all pairs of features is time consuming, scaling quadratically with the number of features and linearly with the size of the tree. Scaling EvolCCM to thousands of features and thousands of genomes will be infeasible; however, we can greatly expand its applicability by aggressively reducing inferred feature sets to those of greatest interest to the user. With this in mind, we have developed ParallelEvolCCM, an R wrapper that supports many types of feature filtering, uses parallelization to accelerate the generation of results, and provides a command-line interface that allows the user to invoke EvolCCM automatically. We demonstrate the utility of ParallelEvolCCM and its parameters by applying it to 1000 genomes from the genus *Bifidobacterium*, a group of Gram-positive, nonmotile bacteria with relatively small genomes and high GC content, which encompasses a diversity of species and strains that are associated with health and disease.

## Methods

ParallelEvolCCM uses the EvolCCM R library from Liu et al. (2023). All phylogenetic operations are performed using the ape library (Paradis and Schliep 2019). EvolCCM models the transition rates of a set of binary features as a combination of their intrinsic rates and the association strength between features during the evolutionary process. Although the states in the original paper were based on the presence or absence of genes (i.e. phylogenetic profiles), EvolCCM can compare any set of features that have binary presence/absence states.

Given a vector $S_{i}=\{x_{i,k},\;k=1,\;\ldots ,\;n\}$ representing the system in state *i*, where $x_{i,k}$ denotes the state of a feature *k* among *n* total features, the instantaneous transition rate $\tau_{i,k}$ for the specific gene *k* is defined as


$$\log(\tau_{i,k})=\;\alpha_{k}-\;\beta_{kk}x_{i,k}-\;\overset{n}{\underset{h\neq k}{\sum }}\;\beta_{hk}x_{i,k}x_{i,h},$$

where $\alpha_{k}$ represents the intrinsic rate for feature *k*; $x_{i,k}$ and $x_{i,h}$ denote the current states of feature *k* and another feature *h* in the system, with values of −1 for absence and 1 for presence; $\beta_{kk}$ indicates half the difference between the gain and loss rates of feature *k*; and $\beta_{hk}$ is the coefficient of interaction between features *k* and *h*. The evolutionary changes in the system state along a phylogenetic tree are modeled as a continuous-time Markov process and the likelihood function *L* across the tree is constructed using Felsenstein’s pruning algorithm (Felsenstein 1973). The maximum-likelihood estimates of the parameters in the transition rates are then obtained using the quasi-Newton method (Paradis and Schliep 2019).

The estimated parameters provide insights into the evolutionary dynamics of the features. The intrinsic rate $\alpha_{k}$ represents the natural evolutionary base rate for feature *k*, and the interaction term $\beta_{hk}$ demonstrates the strength and direction of the association between two features. To test the significance of these associations, a *Z*-test is performed to compute a *P*-value for the null hypothesis $H_{0}$: $\beta_{hk}$ = 0 (i.e. that the two features have no association with one another).

### Input

ParallelEvolCCM accepts as input a tab-separated feature file, where each row corresponds to a genome and each column a feature. “0” in a given row/column combination indicates the absence of the corresponding feature from a genome, and “1” its presence. The script also requires a Newick-formatted tree as input. EvolCCM requires a rooted tree: if the user-provided tree is unrooted, ParallelEvolCCM performs a midpoint rooting of the tree. All genome IDs specified in the feature file must have corresponding leaves in the tree; however not all leaf labels need to be present in the feature file. By default any multifurcations in the input tree are resolved randomly; a hardcoded option in the script can be changed to end execution if the provided tree is not binary.

### Parallelization

Parallelization is controlled through the “–cores” option. Specifying a positive integer will attempt to use the corresponding number of CPU cores for the pairwise gene computations. Specifying “−1” as a value will attempt to use all available cores on the system. If no value is specified, EvolCCM will use only a single core.

### Feature Filtering

ParallelEvolCCM optionally uses two techniques to perform feature filtering. Features can be removed from consideration based on their abundance, with the logic that features that are found in all or nearly all genomes are unlikely to be interesting from a modeling point of view, and rare features will in most cases have minimal overlap in occurrence patterns, yielding no informative associations. Minimum and maximum abundance thresholds can be set independently, with any feature outside these thresholds removed from further consideration.

Subsets of features can be identified with common prefixes: for example, all plasmid-based annotations might be prefixed with “plasmid_,” or all genes with a given Gene Ontology biological process ontology term with the ID of that term. Two flags, “–compare_from” and “–compare_to,” allow the user to define subsets of the full feature set for comparison; each defaults to the full set. For example, if the full set is comprised of 100 features, 20 of which are prefixed with “plasmid_” and 10 with “genomicisland_,” then running ParallelEvolCCM without the two flags will perform all $\left(\begin{array}{c} 100\\ 2 \end{array}\right)$ = 4950 pairwise comparisons. Specifying “–compare_from plasmid_, genomicisland_” will compare only features with those prefixes against the full set of features, for a total of $\left(\begin{array}{c} 100\\ 2 \end{array}\right)$ − $\left(\begin{array}{c} 70\\ 2 \end{array}\right)$ = 2535 comparisons, removing all comparisons among the 70 features not in the “compare_from” set. Similarly, “–compare_from plasmid_ –compare_to plasmid_” will perform $\left(\begin{array}{c} 20\\ 2 \end{array}\right)$ = 190 comparisons only within this group of features. ParallelEvolCCM does not perform multiple-test or false-discovery rate corrections; if the user wishes to apply these, it is essential to account for the different number of tests performed under different filtering conditions.

### Program Output and Visualization

ParallelEvolCCM provides detailed standard output about dataset dimensions, cores used, tree operations, and statistics about the number of filtered features. The tree used for rate inference after any midpoint rooting or resolution of multifurcating nodes is saved in an output file. Z-scores and *P*-values for all pairwise feature comparisons are output as tab-separated matrices in two separate files. A script, “PECCM_Build_GraphML.py” can be used to generate GraphML files from either of the two matrices, for import into software packages such as Cytoscape (Shannon et al. 2003).

## Results

We retrieved 1000 assembled genomes assigned to the genus *Bifidobacterium* from the “AllTheBacteria” dataset (Hunt et al. 2024) that had a minimum N50 of 50,000 nucleotides and a length between 1.5 and 3.0 megabases, reflecting the approximate size bounds for the genus (Rodriguez and Martiny 2020). From this larger set, we built a subset of 1000 genomes with species distributions (Table 1) that maintained some level of overrepresentation in the original dataset; for example, *Bifidobacterium longum* remained the most-common genome in the set. *Bifidobacterium* is often associated with positive gut health and dominates the gut microbiota of healthy, breast-fed infants (Arboleya et al. 2016), but the genus also includes species such as *Bifidobacterium vaginale* (recently renamed from *Gardnerella vaginalis*, previously *Corynebacterium vaginale*, previously *Haemophilus vaginalis*: [Catlin 1992]) that are associated with negative health outcomes such as bacterial vaginosis (e.g. Morrill et al. 2020). We also generated a subset of 100 genomes from the 1000-genome set.

**Table 1:** Distribution of most-common species in the 1000- and 100-genome Bifidobacterium datasets

Species name	B1000	B100
*Bifidobacterium longum*	300	25
*Bifidobacterium adolescentis*	150	22
*Bifidobacterium pseudocatenulatum*	100	7
*Bifidobacterium breve*	100	10
*Bifidobacterium animalis*	100	8
*Bifidobacterium infantis*	50	7
*Bifidobacterium bifidum*	50	5
*Bifidobacterium vaginale*	40	6
Other	110	10

We used the ARETE annotation and phylogenomics pipeline (https://github.com/beiko-lab/arete) to perform annotation and phylogenomic analysis of both these subsets independently. Distribution information was generated for several types of features: antimicrobial-resistance genes using version 6.0.2 of the Resistance Gene Identifier (Alcock et al. 2023), plasmids using MOB-suite (Robertson and Nash 2018) version 3.0.3, and metal resistance genes and virulence factors through homology search against the BacMet (Pal et al. 2014) and VFDB (Liu et al. 2022) databases using DIAMOND version 2.0.15 (Buchfink et al. 2021). Reference phylogenetic trees were constructed from the concatenated core-genome sequence alignment generated by PPanGGOLiN v2.0.5 (Gautreau et al. 2020) using Fasttree version 2.1.10 (Price et al. 2010). We then ran ParallelEvolCCM using various parameter settings to demonstrate runtimes and outputs. Comparisons between very rare features can sometimes fail to converge, leading to invalid small or large Z statistics; these were removed from the results set prior to calculation of distributions.

**Fig. 1.**
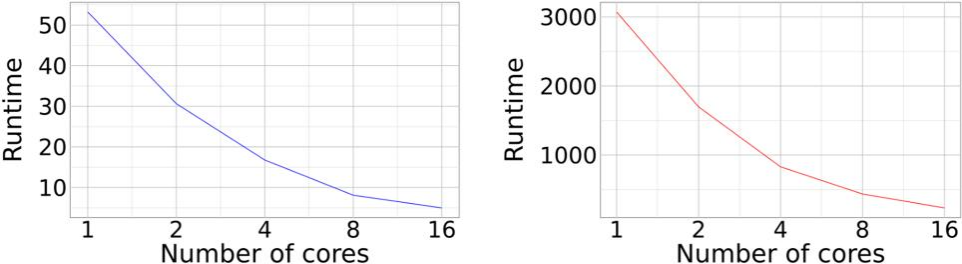
Runtimes in minutes for features extracted from 100 genomes (a) and 1000 genomes (b) with different numbers of allocated cores.

Parallelization of comparisons into concurrent tasks showed its effectiveness, as demonstrated in Fig. 1. Each twofold increase in the number of cores yielded a speedup factor between 1.6 and 2.1, with the 100-genome run requiring between 5 and 54 min, and the 1000-genome run requiring a minimum of 235 min and a maximum of 3072.

**Fig. 2.**
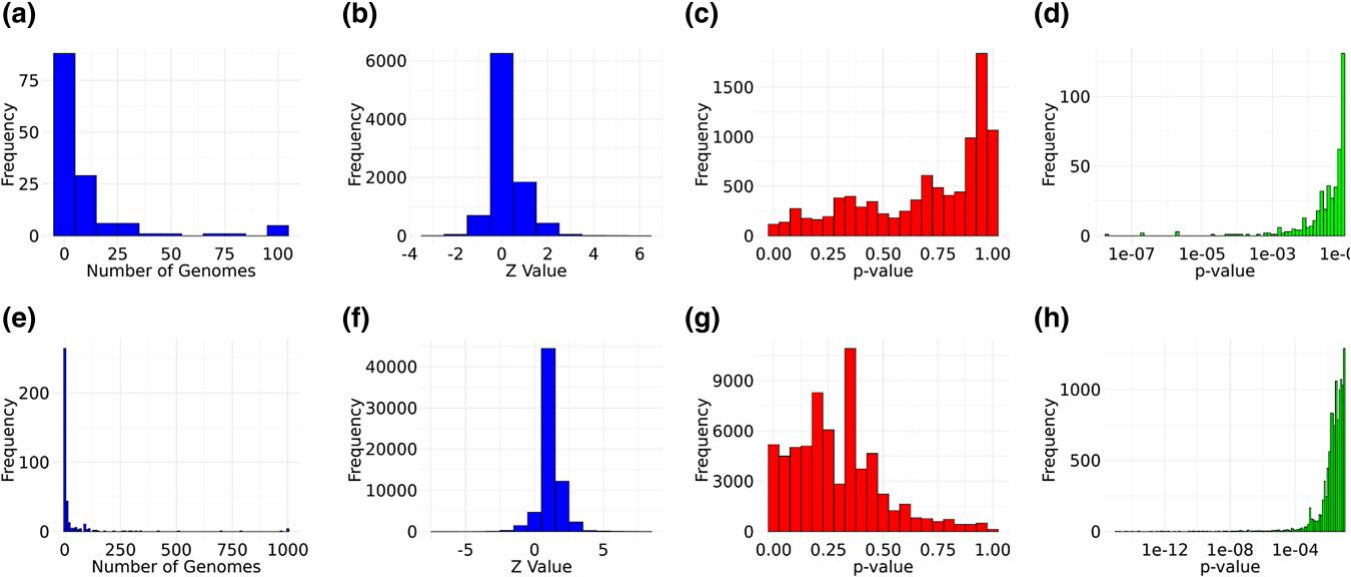
Feature and statistical score distributions for 100-genome (a–d) and 1000-genome (e–h) datasets. a/e) Frequency distribution of feature counts across genomes. b/f) Distribution of Z scores across all pairwise feature comparisons. c/g) *P*-value distribution across pairwise comparisons on a linear scale from *P* = 0 to 1. d/h) distribution of *P*-values less than or equal to 0.1 on a logarithmic scale.

Figure 2 shows the feature and statistical distributions for the 100-genome and 1000-genome datasets. A total of 138 features (13 plasmids predicted by MOB-suite, 115 AMR genes predicted by RGI, 2 biocide/metal resistance genes predicted by BacMet, and 8 virulence-factor genes predicted by VFDB) were observed at least once in the 100-genome dataset (Fig. 2a); however, only 11 of the 115 predicted AMR genes had Perfect and/or Strict matches. The 1000-genome dataset had a total of 384 predicted features (40 plasmids predicted by MOB-suite, 317 AMR genes predicted by RGI of which 23 had Perfect and/or Strict matches, 8 biocide/metal resistance genes predicted by BacMet, and 19 virulence-factor genes predicted by VFDB); 333 of these were present in fewer than 50 genomes and 256 in fewer than five (Fig. 2e). Interaction Z scores in the 100-genome dataset ranged from −2.47 to 5.60, and the smallest *P*-value of $2.2\;\times \;10^{-8}$ (Fig. 2b–d) was obtained from the *ugd* gene, which is found in both the RGI and VFDB predictions; this score was obtained in spite of incomplete agreement between the predictions derived from CARD and VFDB which use different thresholding approaches to assign genes to specific classes. The comparison between *ugd* and the *rpoV* sigma-factor gene yielded the largest negative Z score (−2.4688) with a corresponding *P*-value of 0.014; given the number of pairwise tests carried out, this relationship is unlikely to be statistically or biologically significant.

The 1000-genome dataset (Fig. 2f–h) had 37 feature pairs with an associated *P*-value of 0, with a corresponding range of Z scores between −63,539 and 255,053; the remaining feature comparisons had Z scores that ranged between −4.24 and 7.37. Thirty-six of the 37 pairs with *P*-values of 0 all included one or more of plasmid AC611, or the *ugd* and *oleI* AMR genes. These three features always had interaction scores of high magnitude or 0, suggesting that their distributions may lead to a failure of the EvolCCM function to converge. The smallest nonzero *P*-value was $1.69\;\times \;10^{-13}$ for the comparison between the beta-lactamase gene *cfxA* and the streptothricin-resistance determinant *sat*-3. The smallest nonzero *P*-value for a negative association was $2.26\;\times \;10^{-5}$, obtained from a comparison of two genes encoding fluoroquinolone-resistance factors: the *gyrA* gene, which encodes the A subunit of DNA gyrase, and *nfxB*, a negative regulator of the MexAB–OprM and MexCD–OprJ efflux-pump systems (Higgins et al. 2003). Performance when different values of the “–compare_from,” “–min_abundance,” and “–max_abundance” flags is shown in Table 2. Restricting the 1000-genome dataset analysis to features with abundance between 5% and 95% required less than 26 min when eight CPU cores were used; even removing the features that were found in only one or all but one of the genomes yielded significant speedups. Comparing functional subsets of the features to the full set yielded significant speedups, especially for very small plasmid and virulence-factor datasets.

**Table 2:** Summary of runtimes and interactions identified for different subset comparisons

Size	From set	Count	Set sizes	Time (m)	Count (*P* < 10^−3^)	Count (*P* < 10^−5^)
100	All	5 to 95	48/48	7	7	1
100	All	2 to 98	76/76	14	15	2
100	AMR	2 to 98	5/76	3	11	1
100	Plas	2 to 98	5/76	2	2	1
100	VF	2 to 98	6/76	<1	1	0
1000	All	50 to 950	46/46	26	42	17
1000	All	2 to 998	208/208	564	337	74
1000	AMR	2 to 998	187/208	438	303	66
1000	Plas	2 to 998	16/208	59	33	7
1000	VF	2 to 998	11/208	3	1	1

The 100-genome and 1000-genome datasets were run with different functional subsets of all features (e.g. predicted AMR genes) compared against the entire feature set. Only features with counts in the range shown in “Count” were considered. “Sizes” shows the number of features in the chosen subset and the full set, once the Count restrictions were applied. Runtime in minutes, and the number of interactions identified at two different *P*-values are shown. All runs described in this table were assigned eight CPU cores.

**Fig. 3.**
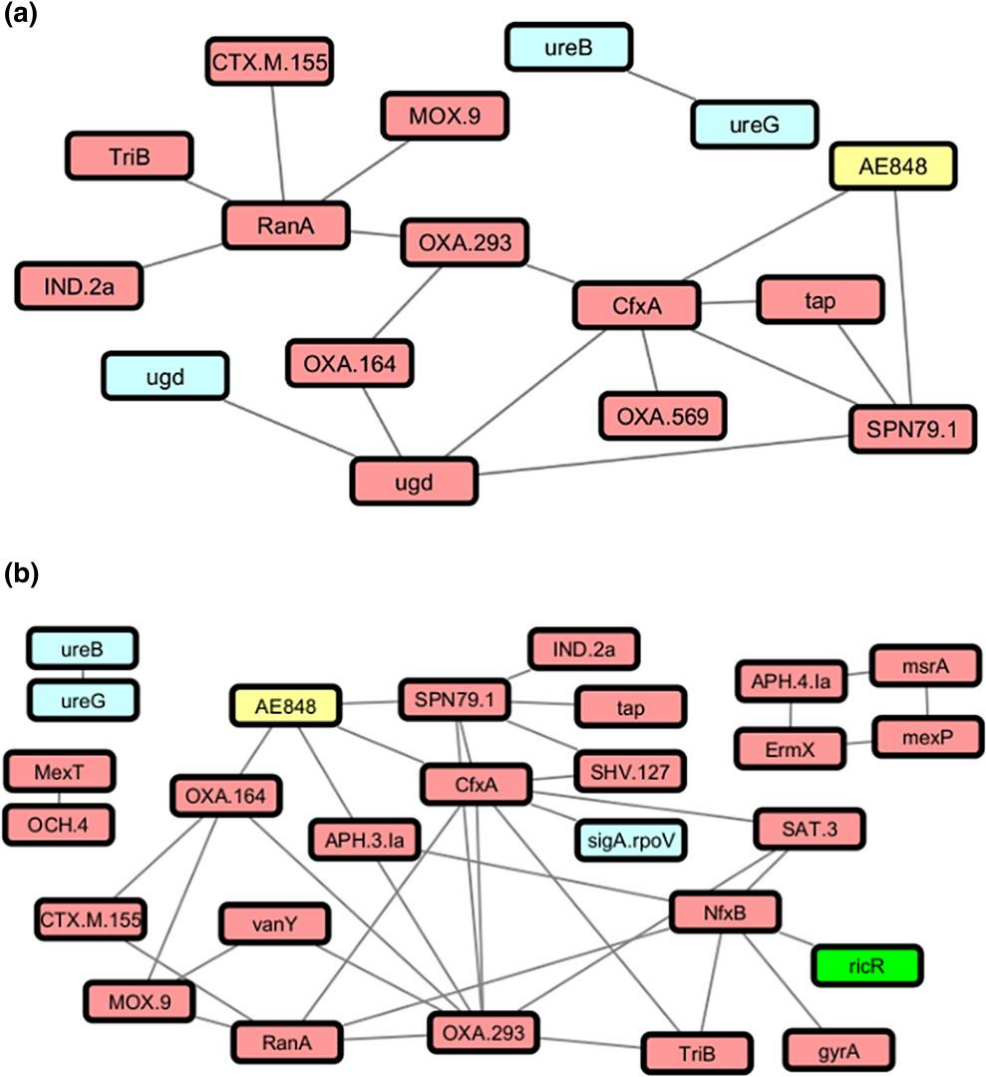
Network visualizations of associations inferred using EvolCCM. Nodes represent annotated plasmids (yellow), AMR genes (red), VFDB (cyan), and BacMet genes (green) annotated through homology search. a) Connections between features in the 100-genome dataset with associated *P*-values less than 0.005. b) Connections between features in the 1000-genome dataset with associated *P*-values less than 0.0005.

Figure 3 shows thresholded EvolCCM networks visualized in Cytoscape. Thresholding the features in the 100-genome data set at a *P*-value of 0.005 (Fig. 3a) yielded a total of two connected components: one comprising two features (*ureB* and *ureG*) from VFDB, the other containing 12 predicted AMR genes, one predicted plasmid, and one predicted virulence factor. *ugd* is present in both VFDB and CARD, and the two features were connected to one another. The plasmid cluster AE848 is connected to the resistance genes *cfxA* and *spn79-1*; however, both of these predictions are Loose hits according to RGI, their dissimilarity from the reference sequences may indicate a different function, or a divergence due to a relatively large evolutionary distance from the closest reference sequence in CARD. AE848 is based on a single 10.2 KB reference plasmid, *B. longum* pNAC3, in NCBI RefSeq with accession NC_004768 (Corneau et al. 2004). A more-detailed investigation of the contigs assigned to this plasmid cluster could reveal whether the Loose hits are physically present on the plasmid.

Nearly all features in the 1000-genome dataset were in a single connected component containing 19 features, with three additional components comprising the *ureB* and *ureG* genes; *mexT* and *OCH-4*; and *mexP*, *msrA*, *APH(4)-1a*, and *ermX* features respectively (Fig. 3b). Plasmid cluster AE848 was again the only MOB-suite prediction in the filtered graph, with connections to *cfxA* and *spn79-1* as was previously the case, and new connections to *oxa-164* and *aph(3′)-Ia*. Although this reference plasmid is too small to contain all the associated genes in the implied EvolCCM network, the plasmid predicted to occur in *Bifidobacterium* may have different properties that could be investigated by inspecting the sequence assemblies in this dataset.

## Conclusions

The coevolutionary model of EvolCCM performs comparisons of feature profiles with appropriate corrections for phylogenetic correlations. The need for this correction is highest when dataset sampling is uneven, which is frequently the case in intensively sampled species that may contain one or more sets of highly similar outbreak isolates. ParallelEvolCCM accepts tables of features generated from any source and offers a range of options to accelerate computations through parallelization and reduction in the number of pairwise comparisons that need to be done. We previously demonstrated that the “Community” aspect of EvolCCM, which allows comparisons of sets of more than two features at a time, can effectively filter out false-positive connections. Considering sets of size >2 increases the complexity and number of comparisons, and ParallelEvolCCM offers an opportunity to accelerate these investigations as well.

Although we considered both gene and plasmid predictions in this work, the input to ParallelEvolCCM contained no information about associations between the two, i.e. if a given gene was localized to a plasmid or the main chromosome for each genome. One way to present this information would be to perform a mapping of genes to contigs prior to running ParallelEvolCCM; this information could be presented to the software as two separate columns (and potentially a third if the complete gene presence/absence pattern is included as well). Other refinements such as specific nucleotides found at variant sites could be presented in a similar manner once identified using other applications.

## Data Availability

EvolCCM and ParallelEvolCCM are released under the MIT license. The source code for ParallelEvolCCM is available at https://github.com/beiko-lab/arete/blob/master/bin/ParallelEvolCCM.R. Documentation for ParallelEvolCCM can be found at https://beiko-lab.github.io/arete/evolccm/. An archive containing the input files, documentation, ParallelEvolCCM results, and helper scripts used to generate Figs. 2 and 3 are available at the same location as the R script. The feature profiles include the accession numbers for all genomes used in this study.
